# Evaluation of Bonding Behavior between Engineered Geopolymer Composites with Hybrid PE/PVA Fibers and Concrete Substrate

**DOI:** 10.3390/ma17153778

**Published:** 2024-08-01

**Authors:** Yu Ling, Xiafei Zhang, Yanwei Wu, Weiyu Zou, Chuang Wang, Chaosen Li, Wen Li

**Affiliations:** 1 Guangzhou Power Supply Bureau, Guangdong Power Grid Co., Ltd., China Southern Power Grid Co., Ltd., Guangzhou 510620, China; 15915714058@163.com (Y.L.); 18102278577@189.cn (X.Z.); wyw1400@163.com (Y.W.); 13822112199@139.com (W.Z.); 18022327865@163.com (C.W.); 2 School of Civil and Transportation Engineering, Guangdong University of Technology, Guangzhou 510006, China; 2112109088@mail2.gdut.edu.cn; 3 School of Civil Engineering and Transportation, South China University of Technology, Guangzhou 510640, China

**Keywords:** EGC, unoiled PVA fiber, hybrid fiber, bond behavior, cost analysis

## Abstract

Engineered geopolymer composites (EGCs) exhibit excellent tensile ductility and crack control ability, making them promising for concrete structure repair. However, their widespread use is limited by high costs of reinforcement fiber and a lack of an EGC–concrete interface bonding mechanism. This study investigated a hybrid PE/PVA fiber-reinforced EGC using domestically produced unoiled PVA fibers to replace commonly used PE fibers. The bond performance of the EGC–concrete interface was evaluated through direct tensile and slant shear tests, focusing on the effects of PE fiber content (1%, 2%, and 3%), fiber hybrid ratios (2.0:0.0, 1.5:0.5, 1.0:1.0, 0.5:1.5, and 0.0:2.0), concrete substrate strength (C30, C50, and C70), and the ratio of fly ash (FA) to ground granulated blast furnace slag (GGBS) (6:4, 7:3, and 8:2) on interface bond strength. Results showed that the EGCs’ compressive strength ranged from 77.1 to 108.9 MPa, with increased GGBS content significantly enhancing the compressive strength and elastic modulus. Most of the specimens exhibited strain-hardening behavior after initial cracking. Interface bonding tests revealed that a PE/PVA ratio of 1.0 increased tensile bond strength by 8.5% compared with using 2.0% PE fiber alone. Increasing the PE fiber content, PVA/PE ratio, GGBS content, and concrete substrate strength all improved the shear bond strength. This improvement was attributed to the flexible fibers’ ability to restrict thermo–hydro damage and deflect and blunt microcracks, enhancing the interface’s failure resistance. Cost analysis showed that replacing 50% of the PE fiber in EGC with unoiled PVA fiber reduced costs by 44.2% compared with PE fiber alone, offering the best cost–performance ratio. In summary, hybrid PE/PVA fiber EGC has promising prospects for improving economic efficiency while maintaining tensile ductility and crack-control ability. Future optimization of fiber ratios and interface design could further enhance its potential for concrete repair applications.

## 1. Introduction

Concrete is the cornerstone of modern infrastructure, used extensively in buildings, bridges, dams, cement concrete pavement, and other critical structures due to its versatility, strength, and durability [1,2,3,4]. However, concrete structures degrade over time, experiencing issues like cracking, rebar corrosion, and spalling, which compromise their integrity and safety [5,6]. This premature aging of reinforced concrete structures poses significant challenges, threatening public safety, increasing maintenance costs, and potentially straining future economies [7]. To mitigate these problems while preserving the function and integrity of these structures, it is crucial to minimize the frequency and extent of repair interventions. Traditional concrete repair and reinforcement methods typically use supplementary materials to restore or enhance deteriorated structures. These materials include FRP, steel plates, and various concrete repair compounds, applied through bonding, overlaying, spraying, and pressure grouting techniques [8,9,10]. However, the durability of these repair methods is often questioned, with concerns about thermal degradation of epoxy resins and corrosion of steel [11]. As a result, concrete repairs are frequently criticized for their lack of early performance and long-term durability.

Cement-based repair materials are inherently brittle, exhibiting low tensile strength, low strain capacity, and poor ductility. Engineered cementitious composites (ECCs), known for their excellent tensile properties and crack control, offer a solution to these issues [12,13,14,15]. Research has shown the feasibility of using ECCs for repair and reinforcement [16,17,18,19,20]. However, ECCs require significant amounts of ordinary Portland cement, contributing to global CO_2_ emissions, which increases their carbon footprint by at least 5–8% [21]. To address the global challenge of climate change, researchers have proposed using industrial by-products, such as fly ash (FA) and ground granulated blast furnace slag (GGBS), to produce engineered geopolymer composites (EGCs). This approach avoids high-carbon cement materials, reducing the carbon footprint of repair materials [22,23,24,25]. The utilization of EGCs can be adopted as repair materials for the rehabilitation of existing building structures, concrete pavements, etc.

Controlling the cost of repair materials is a crucial issue for the application of EGCs. In EGC-like materials, polyvinyl alcohol (PVA) fibers, polyethylene (PE) fibers, and other polymer fibers like aramid fibers or polyethylene terephthalate (PET) fibers have been adopted for their various benefits [26,27,28,29]. However, frequently used fiber types like oiled PVA fibers or PE fibers are expensive. For example, Kuraray Co., Ltd. in Japan monopolizes the surface-coating technology for PVA fibers, making them accountable for up to 70% of the total material costs. To promote widespread application, reducing preparation costs is essential. One effective method is to partially replace high-performance fibers with more affordable ones such as unoiled domestic PVA fibers, at only 10–20% of the cost of PE fibers.

It was reported that oiled PVA fibers reduced excessive bonding between hydrophilic PVA fibers and the surrounding matrix, achieving the desired tensile performance for PVA-ECC [30]. Nevertheless, although prone to premature rupture due to excessive bonding, unoiled PVA fibers can be suitable for ECC materials when the matrix is customized to compensate for these changes [28,31]. Fortunately, it was reported that the fracture toughness of the EGC matrix was so low that the fiber’s mechanical performance requirements as well as the fiber content could be reduced. In the study by Wang et al. [32], it was found that using 0.1–1.5% PE fibers maintained the high tensile strain capacity of EGC. Thus, it can be inferred that incorporating unoiled PVA fibers can significantly reduce costs while maintaining the high tensile strain capacity of EGC, compared with using PE fibers or oiled PVA fibers alone.

Despite the promising properties of EGCs with hybrid PVA/PE fibers, the bond performance of the EGC–concrete interface deserves attention when it comes to the repairing of concrete structures. The interface bond strength is crucial to the performance of repaired structures. Factors affecting bond strength include the strength grade of the concrete substrate and repair material [18,33], interface roughness, and casting methods [17,34,35]. Existing studies have shown a positive correlation between the strength of the concrete substrate, the repair material, and the interface bond strength [17]. For instance, an improvement in interface shear strength of up to three-fold was reported when the compressive strength of ECC increased from 21.7 MPa to 40.8 MPa [17]. Properly treating the interface to increase roughness can improve bond strength, but overly rough surfaces may be detrimental [33]. Increasing the sand-to-cement ratio can reduce microcracks at the EGC–concrete interface, improving bond strength [36]. However, comprehensive studies on bonding between hybrid fiber EGC and concrete substrates are limited.

Systematic research is urgently required to unravel the unique bonding mechanisms between hybrid fiber EGC and concrete for advanced engineering applications. This study introduces a new approach by utilizing domestically sourced unoiled PVA fibers as a replacement for conventional PE fibers, leading to the development of an innovative hybrid PE/PVA fiber EGC. The bonding performance of the EGC–concrete interface was investigated through direct tensile and slant shear tests, exploring the effects of different fiber hybrid ratios, concrete substrate strength, and FA/GGBS ratios on interface bond strength. Additionally, a cost analysis compared the EGC developed in this study with others reported in the existing studies, discussing the feasibility of replacing PE fibers with domestic unoiled PVA fibers. This research not only advances the understanding of bonding behavior between hybrid PE/PVA fiber EGC and concrete substrate, but also paves the way for its practical application in repair and reinforcement, thereby promoting its widespread adoption in the industry.

## 2. Experimental Program

### 2.1. Materials and Mix Proportion

#### 2.1.1. EGC with Hybrid PE/PVA Fibers

**Figure 1:**
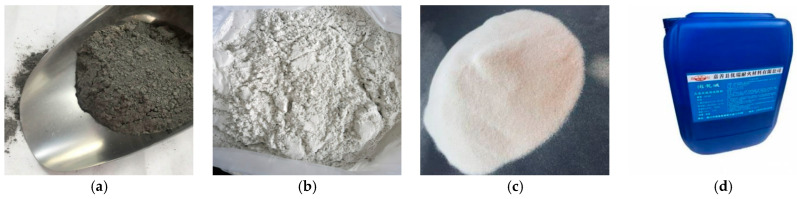

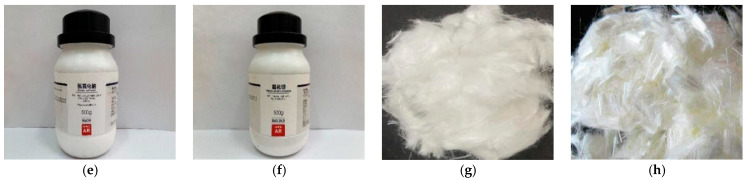
Raw materials of EGC: (**a**) fly ash; (**b**) ground granulated blast furnace slag; (**c**) quartz powder; (**d**) sodium silicate; (**e**) sodium hydroxide; (**f**) barium chloride; (**g**) polyethylene fiber; (**h**) polyvinyl alcohol fiber.

**Figure 2:**
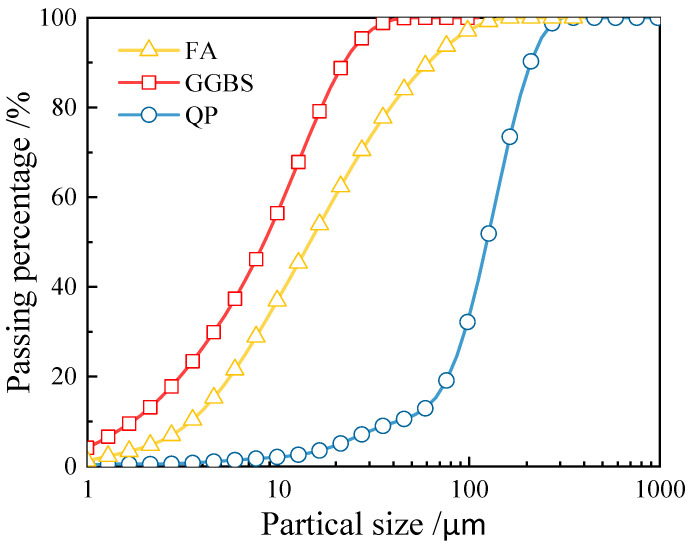
Particle size distributions of raw materials.

Figure 1 shows the raw materials used to prepare the EGC in this study, including Class F FA, S105 GGBS, quartz powder (QP), sodium hydroxide (SH), sodium silicate (SS), polyethylene fibers (PE fibers), domestic unoiled polyvinyl alcohol fibers (PVA fibers), barium chloride (BC), defoamer, and water. Class F FA and S105 GGBS conformed to the requirements of GB/T 51003—2014 [37]. Figure 2 displays the particle size distribution of FA, GGBS, and QP, determined through laser particle size analysis. Table 1 presents the chemical composition of FA and GGBS using X-ray fluorescence (XRF). X-ray fluorescence (XRF) is an analytical technique used to determine the elemental composition of materials, which is based on the principle that individual elements emit characteristic “secondary” (or fluorescent) X-rays when they are excited by high-energy X-rays or gamma rays.

SH and SS were used to prepare the alkali activator. The SS solution had a modulus of 2.25, with a mass ratio of *m*(SiO_2_): *m*(Na_2_O): *m*(H_2_O) = 29.99: 13.75: 52.26. The modulus in sodium silicate is given by the molar ratio of SiO_2_ to Na_2_O, indicating the composition of sodium silicate. The SH solution had a concentration of 10.00 mol/L. The alkali activator was made by mixing SS solution and SH solution in a 2:1 ratio. To prepare the 10 mol/L SH solution, 400 g of SH flakes were dissolved in water to make 1 L of solution, which was then mixed with the SS solution, stirred, sealed, and stored for later use.

BC was used as a retarder to delay the rapid hardening of EGC. Table 2 lists the relevant parameters for PVA and PE fibers. Defoamer was also used to reduce bubbles during mixing. The mix proportions of the hybrid fiber EGC are shown in Table 3. The naming convention for the mix proportions is Mx-UyPz, where x represents FA/GGBS ratios of 7:3, 6:4, and 5:5 (for x = 1, 2, 3, respectively), and y and z represent the volume content of PE fibers and PVA fibers, respectively.

#### 2.1.2. Concrete Substrate

In this study, ordinary concretes with compressive strengths of 30, 50, and 70 MPa were used as the concrete substrate. The mix proportions are detailed in Table 4. The raw materials included water, cement, coarse aggregate (gravel), fine aggregate (river sand), and a water-reducing agent. The cement was 42.5R ordinary Portland cement. The coarse aggregate consisted of 5–10 mm granite gravel, and the fine aggregate was dried river sand. The water-reducing agent, produced by Guangdong Strong Building Materials Co., Ltd., mainly consisted of β-naphthalene sulfonate condensate, with a water reduction rate of approximately 15–20%. The compressive strength of the concrete substrate was measured using 150 mm cubic specimens, based on the national standard GB50081-2019 [38]. The results showed compressive strengths of 35.3 MPa for C30, 51.3 MPa for C50, and 70.6 MPa for C70.

### 2.2. Specimens and Preparation

**Figure 3:**
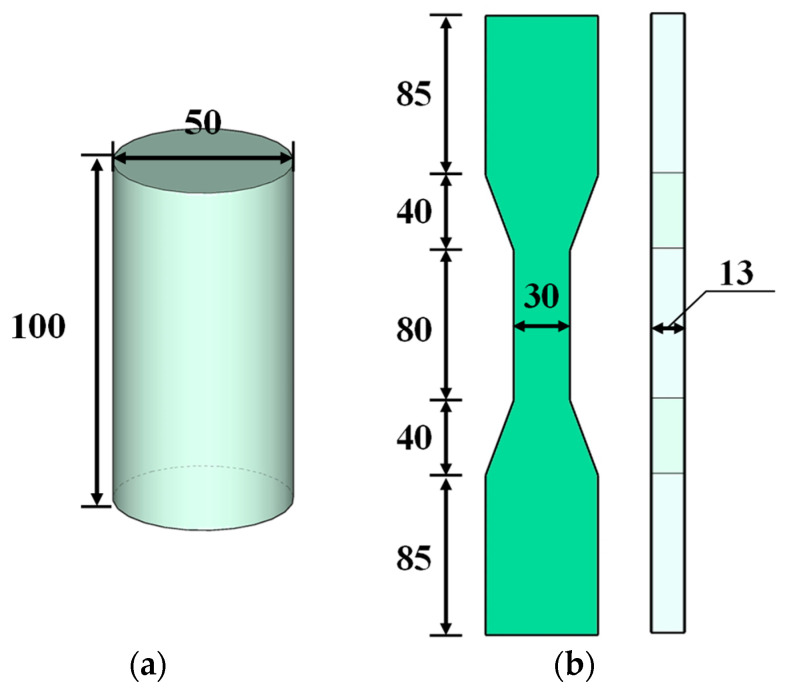
Specimens used for testing the basic mechanical properties of EGC: (**a**) axial compression test; (**b**) axial tension test (dimensions in mm).

**Figure 4:**
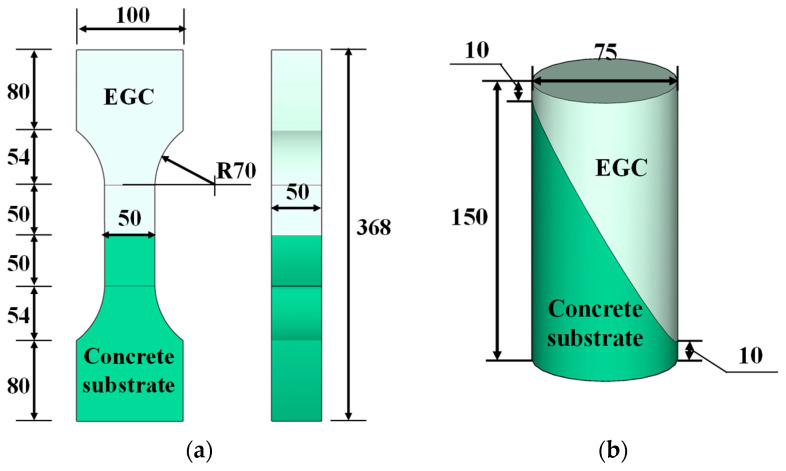
Specimens adopted for bonding behavior between EGC and concrete substrate: (**a**) direct tension test; (**b**) compressive slant shear test (dimensions in mm).

Figure 3 illustrates the specimen dimensions for testing the mechanical properties of hybrid fiber EGC and the concrete substrate. Figure 4 shows the dimensions for the bond performance tests between hybrid fiber EGC and the concrete substrate. In accordance with ASTM C469 [39], a cylindrical specimen with a diameter of 50 mm and a height of 100 mm (Figure 3a) was used to test the axial compression performance of the EGC. A dumbbell-shaped specimen (Figure 3b), per JSCE regulations [40], was used to test the axial tensile performance of the EGC. For bond performance tests, the specimens for direct tension test and slant shear test were designed according to T/CBMF37-2018 [41] (Figure 4a) and ASTM-C882 [42], respectively. Specifically, dumbbell-shaped specimens as shown in Figure 4a were used for direct tension testing, and cylindrical specimens with dimensions of Φ75 mm × 150 mm and an interface inclination angle of 30° were adopted for slant shear tests (Figure 4b). Note that three samples were prepared in each series.

The preparation process of hybrid fiber EGC was as follows: FA, GGBS, BC, and QP were mixed at 76 rpm for two minutes. The prepared solution and water were then added during slow mixing, followed by one minute of medium-speed mixing at 135 rpm. Finally, the fibers were added during slow mixing, which continued for another minute to ensure even distribution. The prepared EGC was placed in molds on a vibrating table for compaction and leveling, covered with plastic film, cured indoors for 24 h, demolded, and cured in water for 28 days.

Table 5 details the specimens used for testing the bond performance between hybrid fiber EGC and the concrete substrate. Specifically, direct tensile tests were conducted to examine the effects of different PE/PVA fiber hybrid ratios on bond performance, keeping the concrete substrate strength grade and FA/GGBS ratio constant. Compressive shear tests investigated the effects of the concrete substrate strength grade, FA/GGBS ratio, and PE/PVA fiber hybrid ratio on bond performance. 

**Figure 5:**
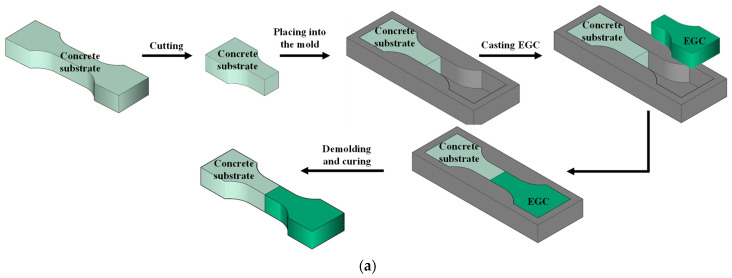

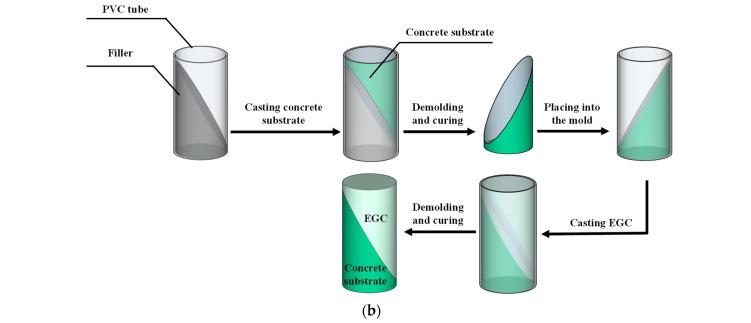
Preparation of specimens adopted for bonding behavior between EGC and concrete substrate: (**a**) direct tension test; (**b**) compressive slant shear test.

Figure 5 depicts the specimen preparation process for bond performance tests. Dumbbell-shaped specimens were prepared by first preparing the concrete side specimens. The mixed concrete was poured into large dumbbell-shaped molds, vibrated, covered with plastic film, demolded after 24 h, and cured in water for 28 days. The concrete specimens were then cut in half, and one half was placed back into the mold, followed by the casting of the EGC. Specimens for slant shear tests required pre-prepared molds. PVC pipes with an inner diameter of 75 mm were cut to a length of 150 mm, and detachable inner cores were 3D printed and placed inside the PVC pipes. The concrete substrate was mixed, cast into the mold, covered with plastic film, and demolded after 24 h of indoor curing. The concrete substrate part of the shear specimen was cured in water for 28 days. Subsequently, the floating slurry at the concrete interface was removed, the concrete part was placed back into the PVC pipe, and the other half of the EGC was cast. After curing indoors for 24 h, the specimens were demolded and cured in water for 28 days until formal testing.

## 3. Experimental Setup and Procedure

### 3.1. Axial Compressive Test

To ensure accurate and reliable measurements, specimens were leveled with high-strength gypsum to avoid eccentric loading. Tests were conducted in displacement control mode with a loading rate of 0.2 mm/min. Axial load was measured using the load cell of the testing machine, while axial strain was measured with strain gauges symmetrically attached to the middle section of each specimen. Axial deformation was monitored by two symmetrically distributed linear variable differential transformers (LVDTs). Data on axial load and deformation were synchronously collected at a frequency of 1 Hz using a static acquisition system.

### 3.2. Axial Tensile Test

The displacement control mode was used with a loading rate of 0.5 mm/min. During the loading process, axial deformation in the central 80 mm region of the specimen was monitored by two symmetrically placed LVDTs.

### 3.3. Test for Assessing Bonding Behavior

For the bond performance tests between concrete and EGC, both direct tensile and shear tests were performed. In the direct tensile tests, universal joints were installed on both sides of the fixtures to prevent eccentric tension, with the loading rate set at 0.2 mm/min, following T/CBMF37-2018 guidelines [41].

In the shear tests, gypsum was applied to the upper and lower surfaces of the specimens to prevent eccentric compression. The loading rate was maintained at 0.2 mm/min in displacement control mode. Load data were collected via the pressure sensor built into the testing machine.

## 4. Results

### 4.1. Compressive Properties of EGC

**Figure 6:**
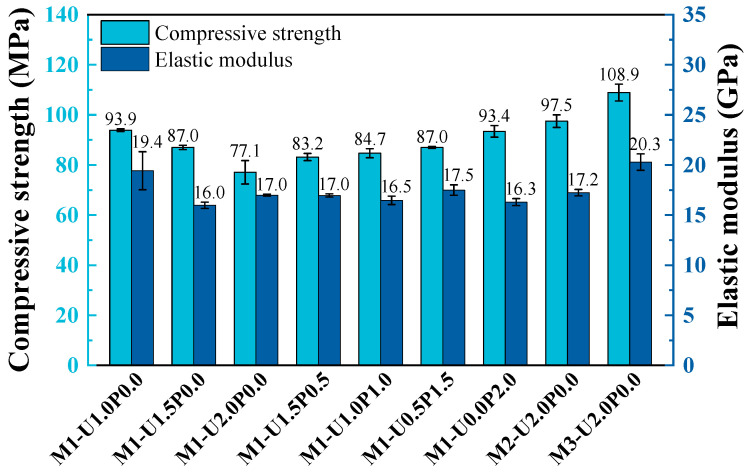
Compressive properties of EGC with hybrid PE/PVA fibers.

Figure 6 illustrates the variations in compressive strength and elastic modulus of hybrid fiber EGCs across different FA/GGBS ratios and hybrid fiber ratios. Overall, the compressive strength and elastic modulus of the prepared hybrid fiber EGC in this study ranged from 77.1 to 108.9 MPa and 16.0 to 20.3 GPa. Specifically, incorporating PE fibers alone resulted in a reduction in compressive strength as the PE fiber content increased. For example, increasing PE fiber content from 1.0% to 2.0% decreased compressive strength by 17.9%. Conversely, substituting PE fibers with PVA fibers led to a consistent increase in the compressive strength of the EGC. In particular, compared with M1-U2.0P0.0 (2.0% PE fibers), M1-U2.0P0.0 (2.0% PVA fibers) exhibited a 21.1% increase in compressive strength. Yet, it was observed that the elastic modulus of the EGC was influenced by PE fiber content and FA/GGBS ratio rather than the fiber hybrid ratio. Furthermore, Figure 6 shows that with identical fiber content, both the compressive strength and elastic modulus of the EGC increased with higher GGBS content. For example, M3-U2.0P0.0 (FA:GGBS = 5:5) exhibited a 16.6% increase in compressive strength and a 24.5% increase in elastic modulus compared with M1-U2.0P0.0 (FA:GGBS = 7:3). 

### 4.2. Tensile Properties of EGC

**Figure 7:**
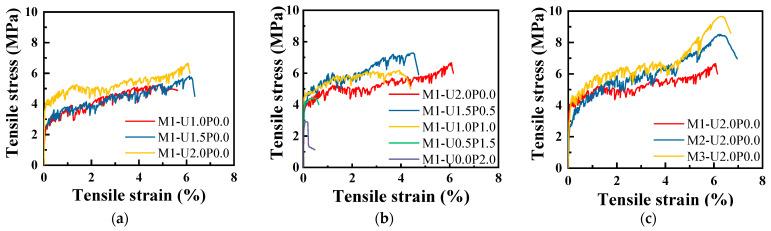
Representative stress–strain curves of EGC with hybrid PE/PVA fibers under axial tension: effects of (**a**) PE fiber content; (**b**) fiber hybrid ratio; (**c**) FA/GGBS.

Figure 7 illustrates representative tensile stress–strain curves of EGC under different variations, highlighting the tensile performance of hybrid fiber EGC under various hybrid PVA/PE fiber ratios and FA/GGBS ratios. Most of specimens demonstrated significant strain-hardening behavior, capable of sustaining loads even after matrix cracking initiated. This behavior is essential for applications requiring high durability and resilience. However, notable exceptions were observed in specimens with higher PVA/PE fiber ratios. These specimens lost their crack-control ability, which is not acceptable in EGC-like materials, transitioning into a strain-softening stage soon after the initial cracking. This loss of tensile performance is crucial as it suggests limitations in the use of high PVA fiber content for certain structural applications. As depicted in Figure 7a, increasing the PE fiber content enhanced the tensile strength and ultimate tensile strain of the EGC, indicating that PE fibers contributed significantly to the material’s load-bearing capacity and tensile ductility. Conversely, it was observed with increasing PVA fiber content, the tensile performance of EGC gradually declined, with the material losing its characteristic tensile properties. Figure 7c indicates that reducing the FA/GGBS (increasing GGBS content) improved tensile performance, suggesting that GGBS played a vital role in improving the overall tensile behavior of the EGC. Overall, hybrid fiber ratios and FA/GGBS ratios were both demonstrated to affect the tensile properties of the EGC, implying that these two essential parameters should be chosen cautiously to preserve excellent tensile properties while saving costs. 

### 4.3. Bonding Behavior

#### 4.3.1. Failure Mode of Specimens in Direct Tension Tests

**Figure 8:**
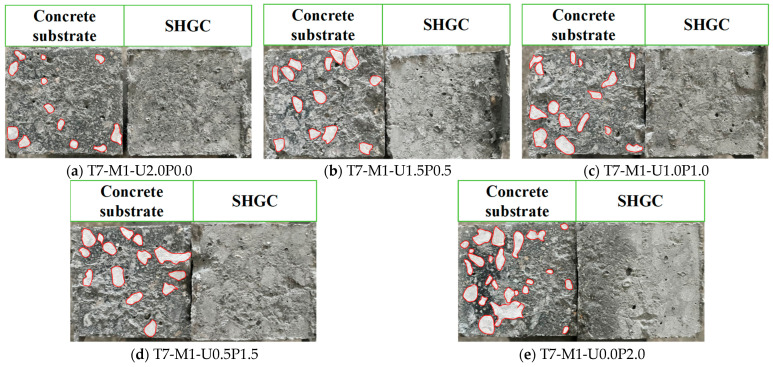
Failure mode of specimens in direct tension tests (the white areas represent the exposed aggregate of the concrete substrate).

Figure 8 depicts the failure modes of specimens in the direct tensile tests on the bond performance of the EGC–concrete interface, with concrete substrate on the left and EGC on the right. It is evident from Figure 8 that the main cracks after failure appeared alternately on both sides of the interface, resulting in the pulling out of both the concrete substrate and the EGC. This phenomenon is commonly referred to in the literature as interface alternating failure [43]. Bonding of EGC to the concrete side was consistently observed across all groups, as shown in Figure 8. Moreover, an increase in the proportion of unoiled PVA fibers correlated with worsened damage on the concrete substrate side, evidenced by an increasing area of exposed aggregate. For instance, in group I7-M1-U0.0P2.0, where the EGC was reinforced with PVA fiber alone, the concrete side exhibited the most prominent exposed aggregate. This observation may be attributed to the relatively minor strength difference between C70 concrete and EGC repair materials. As the proportion of PVA fibers increased from 0% to 100%, the compressive strength of EGC gradually improved, thereby enhancing to some extent the bond strength at the interface.

#### 4.3.2. Direct Tensile Bond Strength

After the failure of the specimens, the peak load at failure was recorded, and the direct tensile bond strength *f*_s_ was calculated using the formula:

(1) $$f_{s}=\frac{p}{A}$$

where *f*_s_ is the direct tensile bond strength (MPa), *P* is the peak load (N), and *A* is the bonding area (50 × 50 = 2500 mm^2^). The results of the bond strength between the EGC repair material and ordinary concrete substrate are presented in Table 6.

Table 6 presents the results of the direct tensile tests for the bond performance of the EGC–concrete interface. It can be observed that substituting PVA fibers for PE fibers slightly increased the tensile bond strength. When PE/PVA = 1.0, the direct tensile bond strength was 8.5% higher than with 2.0% PE fibers alone, indicating that using cost-effective unoiled PVA fibers did not negatively affect the bond performance of the EGC–concrete interface. Instead, it slightly improved the bond strength while saving costs. Since the material lost its characteristic tensile properties when PE/PVA was below 1.0, it is recommended to use M1-U1.0P1.0 (PE/PVA = 1.0) as the repair material for the EGC prepared in this study. This approach slightly enhanced the direct tensile bond strength while maintaining the tensile properties of the EGC and reducing costs. Note that in this study, the concrete substrate surface was not roughened. Given the significant impact of interface roughness, it is suggested to increase the surface roughness to further enhance bond strength.

#### 4.3.3. Failure Mode of Specimens in Compressive Slant Shear Test

**Figure 9:**
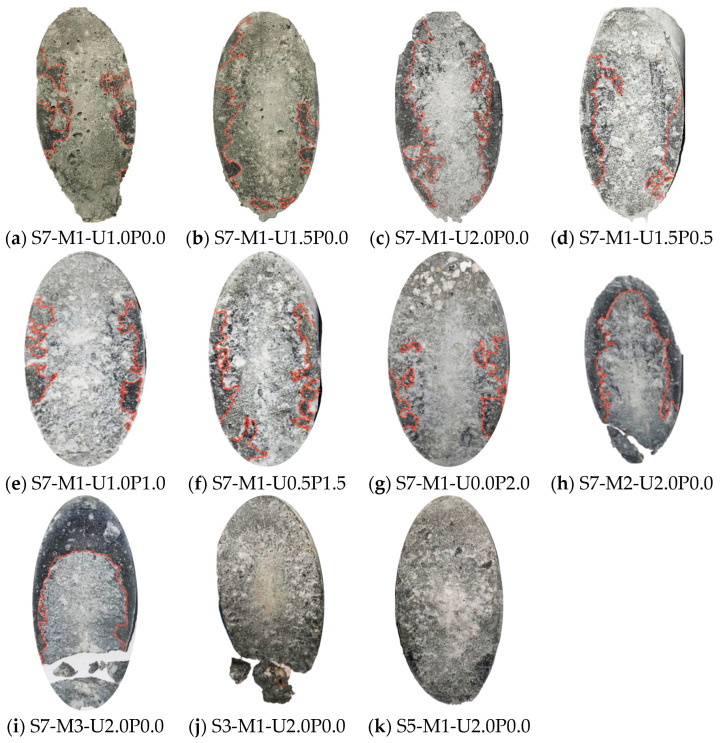
Failure mode of specimens in the compressive slant shear test (concrete substrate side with the shaded area representing EGC).

Figure 9 illustrates the failure modes of the interface specimens on the concrete substrate side in the compression shear test, with dark-colored areas representing the concrete substrate. Despite showing only the concrete substrate side after specimen failure, it was evident that the concrete substrate side and EGC side of specimens maintained fundamentally good integrity. However, fractures in the concrete substrate occurred when there was a significant strength difference between the concrete substrate and the repair EGC. Integrity is defined as the condition of the concrete substrate and repair material; cracks or fractures in either indicate poor integrity. The integrity of both sides also reflects the sequence of failure at the bonding interface. Good integrity suggests that damage first occurs at the interface. Thus, as the bonding performance improves, the integrity of the substrate and repair mortar decreases. In this study, the fractures observed on the concrete substrate side of S7-M2-U2.0P0.0, S7-M3-U2.0P0.0, and S3-M1-U2.0P0.0 indicated relatively good bonding performance, consistent with the calculated results. Therefore, it was concluded that the EGC showed better repair effects in the process of concrete rehabilitation, which was likely to have been due to the increased tensile strength of the EGC, leading to improved bonding performance. 

#### 4.3.4. Shear Bond Strength

As per ASTM C882 [42], failure loads in the slant shear test were recorded. The shear bond strength was calculated by dividing the load at failure by the bonded area. The specific calculation formula was as follows:

(2) $$\tau_{n}=\frac{P}{A}$$

where *P* is the peak load (N), and A is the elliptical bonded area of the cylindrical specimen as specified by ASTM C882 (9116 mm^2^) [42]. To eliminate the impact of size, this study ensured that the repair interface area of each sample was the same. The shear bond strength of the specimens was calculated to analyze the effects of PE/PVA fiber hybrid ratios, FA/GGBS ratios, and PE fiber content on the EGC–concrete shear interface performance. The calculated results are shown in Table 7.

**Figure 10:**
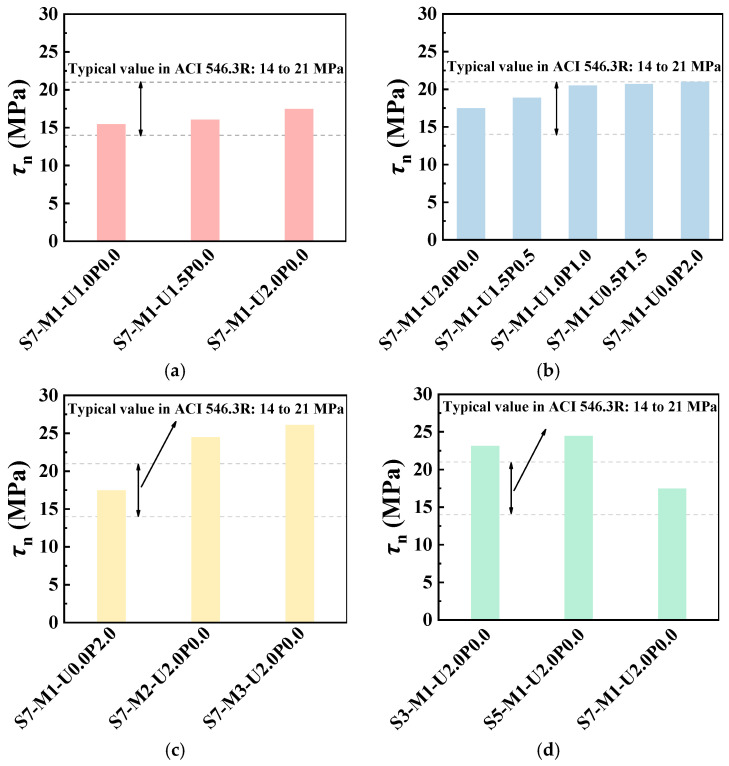
Interfacial shear bond strength between EGC and concrete substrate based on compressive slant shear test. Effects of (**a**) PE fiber content; (**b**) PE/PVA fiber hybrid ratio; (**c**) FA/GGBS; (**d**) concrete substrate strength grade.

Figure 10 presents the effects of various variables on the shear bond strength. From Figure 10, it is evident that PE fiber contents, fiber hybrid ratios, FA/GGBS ratios, and the concrete substrate strength all influenced the shear bond strength to varying degrees. The shear bond strength generally met or exceeded the range recommended by ACI 546.3R [44], a guide to materials selection for concrete repair reported by the American Concrete Institute (ACI) committee 546. 

As the PE fiber content increased, the shear bond strength increased as well. For instance, with PE fiber contents of 1% (S7-M1-U1.0P0.0), 1.5% (S7-M1-U1.5P0.0), and 2% (S7-M1-U2.0P0.0), the shear bond strengths were 15.5 MPa, 16.0 MPa, and 17.5 MPa, respectively, which were 10.7%, 14.3%, and 25.0% higher than the minimum standard of ACI 546.3R [44]. Additionally, when the PE fiber content of EGC was 2.0%, the shear bond strength increased by 3.2% and 12.9% compared with that with 1.0% and 1.5% PE fiber content, respectively. Similarly, increasing the PVA fiber hybrid ratio, GGBS ratio, and concrete substrate strength grade all exerted positive influence on the shear bond strength. However, for the C70 concrete substrate, the shear bond strength was lower than that of specimens with C30 and C50 concrete substrates. 

### 4.4. Cost Analysis

Table 8 shows the costs of raw materials used in hybrid fiber EGCs, based on bulk commodity prices for laboratory-purchased raw materials. Note that the prices for laboratory-purchased raw materials are generally higher in terms of purchasing amount and shipping costs. Table 9 compares the hybrid EGC mix proportions studied here, focusing on key axial compression and tension performance parameters (compressive strength $\sigma_{c}$, tensile strength $\sigma_{tu}$, ultimate tensile strain $\varepsilon_{tu}$) as economic indicators. The impact of different PVA/PE ratios on the economic efficiency of EGCs with hybrid fibers was analyzed, with the price of oiled PVA fibers sourced from reference [45]. Cost comparisons with EGCs from other studies were also conducted [27,46,47]. However, shear bond strength data are not included in the reported comparison because variations in casting methods, interface roughness, and test methods significantly affected the bonding performance of the EGC–concrete interface.

**Figure 11:**
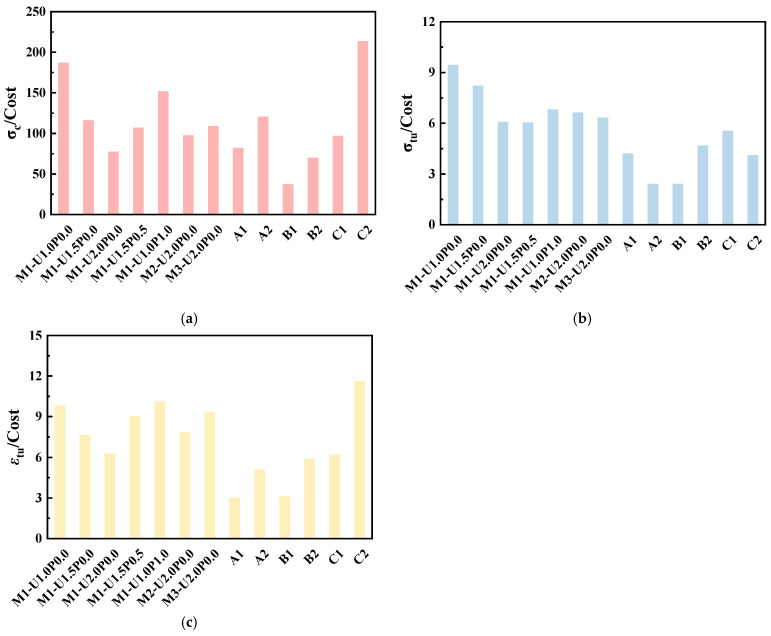
Cost analysis: (**a**) $\frac{\sigma_{c}}{Cost}$; (**b**) $\frac{\sigma_{tu}}{Cost}$; (**c**) $\frac{\varepsilon_{tu}}{Cost}$.

Table 10 displays the mechanical performance of EGCs in this study and other existing research, along with the results of the cost analysis. Figure 11 contrasts these cost analyses, with the cost per cubic meter of M1-U2.0P0.0 set as the baseline (1.0). Note that A3, B3, and C3 were excluded from Figure 11 because of their low tensile capacity. Notably, using PP fibers, domestically produced unoiled PVA fibers, or SFs effectively reduced the cost, but a high substitution rate compromised the material’s excellent tensile ductility, which is crucial for EGCs. From Table 10, it is evident that M1-U2.0P0.0, M2-U2.0P0.0, and M3-U2.0P0.0 incurred the highest costs. The influence of different FA/GGBS ratios on costs was minor but these ratios did affect mechanical performance. From a cost-effectiveness standpoint, increasing the PE fiber content was found to be economically inefficient in this study. 

## 5. Discussion

### 5.1. Effects of Hybrid Fiber Ratios and FA/GGBS Ratios on Compressive Properties of EGCs 

Single incorporation of PE fibers in EGCs was demonstrated to decrease the compressive properties, different from when PVA fibers were utilized to replace PE fibers. In effect, this finding aligned with Yun [48], who reported that uneven dispersion of high-aspect-ratio PE fibers in mortar weakened fiber–aggregate interfacial sections, resulting in premature compressive failure [48]. Additionally, hydrophobic PE fibers may increase entrapped air content and localized porosity during mixing, which could affect the compressive strength and elastic modulus under varying PE fiber contents. Regarding the FA/GGBS ratio, it was found that higher compressive properties could be acquired with lower FA/GGBS (i.e., higher GGBS content). This enhanced performance with increased GGBS content was attributed to the formation of different reaction products. With higher GGBS content, the elastic modulus of C-A-S-H gel ranged from 12 to 47 GPa [49,50], surpassing the N-A-S-H gel formed by FA activation (4.44–20 GPa) [51]. The presence of C-A-S-H gel significantly enhanced both compressive strength and elastic modulus, as reported in previous studies [52]. Still, it is recommended to be cautious when it comes to the selection of the hybrid fiber ratio and FA/GGBS ratio of EGCs, in that flowability and characteristic tensile behavior should be taken into account as well. 

### 5.2. Effects of Hybrid Fiber Ratios and FA/GGBS Ratios on Tensile Properties of EGCs

Test results showed that EGCs reinforced with various fibers presented diverse tensile properties. Higher single PE fiber content and lower hybrid PVA/PE ratios and FA/GGBS ratios exerted positive influences on the tensile properties of EGC. Optimal hybrid fiber and FA/GGBS ratios are essential for maintaining excellent tensile properties and cost efficiency. The improvement in tensile properties was ascribed to changes in the fiber-bridging strength, fiber/matrix interface performance, and mechanical properties of the matrix under different conditions. Within a certain range, higher PE fiber content, whether hybridized with PVA fibers or not, enhanced fiber-bridging strength, thereby boosting the EGC’s tensile performance. The domestically produced unoiled PVA fibers used in this study possessed mechanical properties inferior to PE fibers. Consequently, PVA fibers forming a reliable bond with the matrix (hydrophilic fibers) may experience premature fracture in high-strength matrices, providing lower fiber-bridging strength compared with PE fibers, thus negatively impacting the EGC’s tensile performance. Moreover, increasing GGBS content results in higher production of C-A-S-H and C-S-H products, facilitating the formation of a dense matrix and improving fiber/matrix interface bonding, thereby enhancing tensile performance [53,54].

### 5.3. Effects of Hybrid Fiber Ratios and FA/GGBS Ratios on Bond Behavior

Since the bond behavior between the EGC and the concrete substrate was assessed through both direct tensile tests and slant shear tests, the variations in bonding behavior and the bonding hypothesis are separately discussed in this section. From the results of the direct tensile tests for bond behavior, it was observed that adopting cost-effective unoiled PVA fibers did not harm the bond behavior. Conversely, the tensile bond strength increased modestly while decreasing the cost of the repair material. In effect, with higher hybrid PVA/PE fiber ratios, the compressive strength of the EGC was gradually enhanced, which has been demonstrated to enhance bond strength [55]. 

As far as shear bond strength is concerned, results showed that higher PE content, PVA/PE ratio, and GGBS/FA ratio were helpful. According to existing studies, the resistance mechanisms of interface shear include interlocking, overriding, and fracturing [56]. Interlocking and overriding are activated by creating a rough interface. Since a smooth interface was utilized in this study, these two mechanisms can be disregarded while focusing primarily on resistance to interface fracture. Additionally, the dowel effect observed in concrete repair materials with steel fibers (SFs) is not applicable to the flexible fibers (PE and PVA fibers) used in this study [56,57]. Therefore, the increase in shear bond strength due to the PE fiber content and the PVA fiber hybrid ratio was attributed to the restriction of thermal and moisture damage by the flexible fibers, and the micro cracks’ deflection and blunting. The former was attributed to repair of the cracks and delamination caused by restrained shrinkage, enhancing damage tolerance and promoting interface integrity and thus improving interface failure resistance. This phenomenon has been documented in existing studies.

Cristina Zanotti [58] observed that instability of the interface between plain concrete and fiber-reinforced concrete occurred after the failure of plain concrete. Apart from the contribution of interface roughness to crack propagation, this phenomenon was ascribed to the repair reinforcement of PVA fibers that facilitated stress redistribution around the main crack and the formation of chemical bonds between PVA fibers and the cement paste. Similarly, previous studies indicated that if the repair material had excellent fracture performance, the interface cracks could be prevented from propagating into the repair material, especially when the repair material was fiber-reinforced [59]. Since the excellent tensile ductility and fracture performance of EGC-like materials have been mentioned in existing studies [25,60,61,62,63], it can be inferred that the improved tensile ductility and fracture performance of the hybrid EGCs, due to the increased PE fiber content, PVA fiber hybrid ratio, and GGBS ratio, led to enhanced bond strength at the EGC–concrete interface, benefiting from the aforementioned mechanisms.

### 5.4. Cost Analysis

As mentioned above, the utilization of unoiled PVA fibers to replace commonly used oiled PVA and PE fibers can maintain the mechanical properties of EGC while saving costs to a greater or lesser degree. In practice, this method can be extended to other fiber types (like steel fiber, aramid fiber, PET fiber, etc.). Different fiber types will result in various fiber-bridging capacities due to their mechanical properties themselves and intrinsic hydrophobic or hydrophilic natures, which is of great importance to EGC based on its design principle [10,15,23,48]. In summary, the selection of fiber type should be cautious. It is necessary to comprehensively take the mechanical properties and cost of EGC into consideration. 

## 6. Conclusions

This study developed hybrid PE/PVA fiber-reinforced EGCs using domestically produced PVA fibers without oil coating as a replacement for commonly used PE fibers. The research focused on investigating the bond performance between the EGC and the concrete through direct tensile tests and slant shear bond tests. Key aspects included exploring the influence of different fiber hybrid ratios, concrete substrate strength grades, and FA/GGBS ratios on interfacial bond strength. Additionally, the study compared and analyzed the cost of the EGCs prepared in this research with the selected published literature, discussing the feasibility of replacing PE fibers with domestically produced PVA fibers without oil coating. The conclusions drawn from this study can be summarized as follows:

(1) The compressive strength of EGC ranged from 77.1 to 108.9 MPa. Increasing the PE fiber content decreased the compressive strength, while substitution with PVA fibers led to improvements of up to 21.1%. The elastic modulus was influenced by the PE fiber content and FA/GGBS ratio rather than the fiber hybrid ratio, ranging from 16.0 to 20.3 GPa. When the FA/GGBS was 5:5, the compressive strength and elastic modulus of EGC increased by 16.6% and 24.5%, respectively, compared with when FA/GGBS was 7:3. This improvement was attributed to the increased formation of C-A-S-H gel, which positively affected axial compression performance.

(2) Most specimens exhibited strain-hardening behavior after initial crack formation. Increasing the PE fiber content improved the tensile strength and ultimate tensile strain of EGC. However, as the PVA/PE fiber ratio increased, the tensile performance gradually decreased, with the material completely losing its tensile performance characteristics at a PVA/PE ratio of 1:1. Increasing the GGBS content also contributed to improved tensile performance by altering the fiber-bridging strength, fiber/matrix interface properties, and mechanical properties of the matrix. PVA fibers exhibited lower mechanical performance than PE fibers, potentially leading to premature fracture in high-strength matrices. Conversely, the C-A-S-H and C-S-H gel resulting from GGBS enhanced the matrix densification and fiber/matrix interface bonding, thereby improving tensile performance.

(3) Substituting PVA fibers for PE fibers slightly increased the direct tensile bond strength between EGC and concrete substrates, particularly at a PE/PVA ratio of 1.0, which increased bond strength by 8.5% compared with the inclusion of 2.0% PE fibers only. Slant shear test results showed that increasing the PE fiber content, PVA fiber hybrid ratio, GGBS ratio, and concrete substrate strength all enhanced the shear bond strength. The contributions of PE fiber content and PVA fiber hybrid ratio to the increase in shear bond strength were due to the restrictive effect of flexible fibers on thermal and moisture damage, as well as the enhancement of interface fracture resistance through microcrack deflection and blunting, thereby increasing the interface bond strength. 

(4) The costs of M1-U2.0P0.0, M2-U2.0P0.0, and M3-U2.0P0.0 were the highest, with negligible effects of different FA/GGBS ratios on costs but significant impacts on mechanical performance. From a cost-effectiveness perspective, increasing the PE fiber content was not economical. When the PVA/PE ratio was 1.0, costs decreased by 44.2%, with good cost-effectiveness. However, when the PVA/PE ratio was higher than 1.0, tensile performance significantly decreased despite the decreasing costs. The cost-effectiveness of the EGC developed in this study was relatively superior under the same fiber substitution ratio conditions. Considering both axial compression performance, tensile performance, and cost, M1-U1.0P1.0 is recommended as the preferred repair material.

(5) EGCs with hybrid PE/PVA fiber have promising application prospects as a material for repairing concrete, particularly in enhancing economic efficiency while maintaining excellent mechanical performance. Optimizing the substitution ratio of PE/PVA fibers significantly reduced costs while preserving tensile performance and interface bond strength. Future research can explore the micro-mechanisms of different fiber hybrid ratios on mechanical performance to optimize fiber hybrid ratios and enhance interfacial bond strength. Additionally, studying the effects of interface roughness and casting methods on material performance will promote the practical application and dissemination of EGCs in engineering. Further experimental and simulation studies will also aid in understanding their long-term performance and durability, providing more reliable solutions for concrete repair.

## Figures and Tables

**Table 1:** Chemical composition of GGBS and FA (unit: wt%).

	CaO	SiO_2_	Al_2_O_3_	Fe_2_O_3_	MgO	TiO_2_	SO_3_	Others	LOI ^1^
GGBS	34.00	34.50	17.70	1.03	6.01	/	1.64	5.12	0.84
FA	4.01	53.97	31.15	4.16	1.01	1.13	2.20	2.37	4.60

^1^ LOI means loss on ignition.

**Table 2:** Parameters of PVA and PE fiber.

Fiber Type	Length (mm)	Diameter (mm)	Tensile Strength (MPa)	Elastic Modulus (GPa)	Density (g/cm^3^)	Elongation (%)
PVA	12	20	2500	120	0.97	3.7
PE	12	15	1830	40	1.29	6.9

**Table 3:** Mix proportions of EGC.

Mix IDs	Mix Proportion							Volume Fraction/%	
	FA ^1^	GGBS ^2^	QP ^3^	AAS ^4^	Water	BC ^5^	Deformer	PE ^6^	PVA ^7^
M1-U1.0P0.0	0.70	0.30	0.20	0.40	0.06	0.01	0.001	1.0	0.0
M1-U1.5P0.0								1.5	0.0
M1-U2.0P0.0								2.0	0.0
M1-U1.5P0.5								1.5	0.5
M1-U1.0P1.0								1.0	1.0
M1-U0.5P1.5								0.5	1.5
M1-U0.0P2.0								0.0	2.0.
M2-U2.0P0.0	0.60	0.40	0.20	0.40	0.06	0.01	0.001	2.0	0.0
M3-U2.0P0.0	0.50	0.50	0.20	0.40	0.06	0.01	0.001	2.0	0.0

^1^ fly ash; ^2^ ground granulated blast furnace slag; ^3^ quartz powder; ^4^ alkali-activated solution; ^5^ barium chloride; ^6^ polyethylene fibers; ^7^ polyvinyl alcohol fibers.

**Table 4:** Mix proportions of concrete substrate.

Mix IDs	Cement	Aggregate	Sand	Water	Water Reducer
C30	1.00	3.56	2.37	0.66	0.000
C50	1.00	3.41	1.92	0.63	0.000
C70	1.00	1.75	0.98	0.28	0.005

**Table 5:** Details of test specimens for assessment of bonding behavior between EGC and concrete substrate.

Specimen IDs	Testing Method	Substrate Concrete Strength Grade	Repairing Material	FA/GGBS Ratio of EGC	Volume Fraction of EGC/%	
					PE	PVA
T7 ^1^-M1-U2.0P0.0	direct tension	C70	M1-U2.0P0.0	7:3	2.0	0.0
T7-M1-U1.5P0.5	direct tension	C70	M1-U1.5P0.5	7:3	1.5	0.5
T7-M1-U1.0P1.0	direct tension	C70	M1-U1.0P1.0	7:3	1.0	1.0
T7-M1-U0.5P1.5	direct tension	C70	M1-U0.5P1.5	7:3	0.5	1.5
T7-M1-U0.0P2.0	direct tension	C70	M1-U0.0P2.0	7:3	0.0	2.0
S7 ^2^-M1-U1.0P0.0	compressive slant shear	C70	M1-U1.0P0.0	7:3	1.0	0.0
S7-M1-U1.5P0.0	compressive slant shear	C70	M1-U1.5P0.0	7:3	1.5	0.0
S7-M1-U2.0P0.0	compressive slant shear	C70	M1-U2.0P0.0	7:3	2.0	0.0
S7-M1-U1.5P0.5	compressive slant shear	C70	M1-U1.5P0.5	7:3	1.5	0.5
S7-M1-U1.0P1.0	compressive slant shear	C70	M1-U1.0P1.0	7:3	1.0	1.0
S7-M1-U0.5P1.5	compressive slant shear	C70	M1-U0.5P1.5	7:3	0.5	1.5
S7-M1-U0.0P2.0	compressive slant shear	C70	M1-U0.0P2.0	7:3	0.0	2.0
S7-M2-U2.0P0.0	compressive slant shear	C70	M2-U2.0P0.0	6:4	2.0	0.0
S7-M3-U2.0P0.0	compressive slant shear	C70	M3-U2.0P0.0	5:5	2.0	0.0
S3 ^2^-M1-U2.0P0.0	compressive slant shear	C30	M1-U2.0P0.0	7:3	2.0	0.0
S5 ^2^-M1-U2.0P0.0	compressive slant shear	C50	M1-U2.0P0.0	7:3	2.0	0.0

^1^ T7 denotes direct tension test and that the substrate concrete strength grade was C70; ^2^ S3, S5 and S7 denote that the substrate concrete strength grades were C30, C50, and C70, respectively, in the compressive slant shear test.

**Table 6:** Results of bonding behavior between EGC and concrete substrate in direct tension tests.

Specimen IDs	Compressive Strength (MPa)		Peak Load (kN)	Bonding Area (mm^2^)	Average Tensile Bond Strength (MPa)
	EGC	Concrete Substrate			
T7-M1-U2.0P0.0	77.1	70.6	1.165	2500	0.466
T7-M1-U1.5P0.5	83.2	70.6	1.171	2500	0.468
T7-M1-U1.0P1.0	84.7	70.6	1.270	2500	0.508
T7-M1-U0.5P1.5	87.0	70.6	1.371	2500	0.549
T7-M1-U0.0P2.0	93.4	70.6	1.390	2500	0.556

**Table 7:** Test results of slant shear test.

Specimen IDs	Compressive Strength (MPa)		Applied Stress $\sigma_{0}$ (MPa)	$\mathrm{Interfacial}\;\mathrm{Shear}\;\mathrm{Strength}\tau_{n}$ (MPa)
	EGC	Concrete Substrate		
S7-M1-U1.0P0.0	93.9	70.6	31.4	15.5
S7-M1-U1.5P0.0	87.0	70.6	32.4	16.0
S7-M1-U2.0P0.0	77.1	70.6	35.6	17.5
S7-M1-U1.5P0.5	83.2	70.6	38.5	18.9
S7-M1-U1.0P1.0	84.7	70.6	41.4	20.5
S7-M1-U0.5P1.5	87.0	70.6	41.7	20.7
S7-M1-U0.0P2.0	93.4	70.6	43.9	21.0
S7-M2-U2.0P0.0	97.5	70.6	50.0	24.5
S7-M3-U2.0P0.0	108.9	70.6	53.5	26.1
S3-M1-U2.0P0.0	77.1	35.3	46.6	23.1
S5-M1-U2.0P0.0	77.1	51.3	50.1	24.5

**Table 8:** Cost of raw materials.

Materials	FA ^1^	GGBS ^2^	QP ^3^	NaOH	Na_2_SiO_3_	BC ^4^	Deformer	HRWR ^5^	PE ^6^	PP ^7^	SF ^8^	PVA ^9^	PVA@O ^10^
Cost (USD/kg)	0.27	0.34	0.06	2.80	0.42	3.36	3.08	2.66	56.00	1.89	1.68	6.30	56.00 [45]

^1^ fly ash; ^2^ ground granulated blast furnace slag; ^3^ quartz powder; ^4^ barium chloride; ^5^ high-range water reducer; ^6^ polyethylene fibers; ^7^ polypropylene fiber; ^8^ steel fiber; ^9^ polyvinyl alcohol fibers; ^10^ oiled-coating polyvinyl alcohol fibers.

**Table 9:** Mix proportions of EGC with hybrid fiber.

Mix IDs		Mix Proportion							Volume Fraction/%			
	FA	GGBS	QP	NaOH	Na_2_SiO_3_	Water	BC	HRWR	PE	PP	SF	PVA@O
A1 [46]	0.50	0.50	0.00	0.00	0.12	0.45	0.00	0.00	1.50	0.00	0.50	0.00
A2 [46]	0.50	0.50	0.00	0.00	0.12	0.45	0.00	0.00	1.00	0.00	1.00	0.00
A3 [46]	0.50	0.50	0.00	0.00	0.12	0.45	0.00	0.00	0.50	0.00	1.50	0.00
B1 [47]	0.50	0.50	0.20	0.13	0.32	0.02	0.00	0.00	0.00	0.00	0.00	2.00
B2 [47]	0.50	0.50	0.20	0.13	0.32	0.02	0.00	0.00	0.00	1.00	0.00	1.00
B3 [47]	0.50	0.50	0.20	0.13	0.32	0.02	0.00	0.00	0.00	2.00	0.00	0.00
C1 [27]	0.60	0.40	0.23	0.04	0.27	0.08	0.00	0.00	2.00	0.00	0.00	0.00
C2 [27]	0.60	0.40	0.23	0.04	0.27	0.08	0.00	0.00	1.00	0.00	1.00	0.00
C3 [27]	0.60	0.40	0.23	0.04	0.27	0.08	0.00	0.00	0.00	0.00	2.00	0.00

**Table 10:** Relevant mechanical properties and cost analysis of EGCs with hybrid fibers.

Mix IDs	Cost	$\sigma_{c}$ (MPa)	$\varepsilon_{tu}$ (%)	$\sigma_{tu}$ (MPa)	$\frac{\sigma_{c}}{Cost}$	$\frac{\sigma_{tu}}{Cost}$	$\frac{\varepsilon_{tu}}{Cost}$
M1-U1.0P0.0	0.503	93.9	4.74	4.92	186.87	9.43	9.79
M1-U1.5P0.0	0.751	87.0	6.16	5.74	115.81	8.20	7.64
M1-U2.0P0.0	1.000	77.1	6.07	6.26	77.10	6.07	6.26
M1-U1.5P0.5	0.779	83.2	4.70	7.04	106.77	6.03	9.03
M1-U1.0P1.0	0.558	84.7	3.80	5.64	151.67	6.80	10.10
M1-U0.5P1.5	0.338	87.0	0.80	4.19	257.65	2.37	12.41
M1-U0.0P2.0	0.117	93.4	0.52	3.10	799.02	4.45	26.52
M2-U2.0P0.0	1.000	97.5	6.62	7.83	97.49	6.62	7.83
M3-U2.0P0.0	1.000	108.90	6.31	9.29	108.89	6.31	9.29
A1 [46]	0.757	62.00	3.18	2.27	81.92	4.20	3.00
A2 [46]	0.516	62.00	1.24	2.61	120.25	2.41	5.06
A3 [46]	0.274	62.00	0.38	2.97	226.05	1.39	10.83
B1 [47]	1.003	37.24	2.41	3.12	37.13	2.40	3.11
B2 [47]	0.522	36.43	2.44	3.07	69.77	4.67	5.88
B3 [47]	0.041	33.27	3.70	1.96	804.10	89.43	47.37
C1 [27]	1.000	96.63	5.54	6.19	96.65	5.54	6.19
C2 [27]	0.517	110.34	2.12	6.00	213.35	4.10	11.60
C3 [27]	0.035	111.82	0.54	6.26	3233.62	15.62	181.03

## Data Availability

The data presented in this study are available on request from the corresponding author. The data are not publicly available due to confidentiality issues.
